# HPV-Impfung zur Prävention von Genitalwarzen und Krebsvorstufen – Evidenzlage und Bewertung

**DOI:** 10.1007/s00103-021-03316-x

**Published:** 2021-04-13

**Authors:** Vanesa Osmani, Stefanie J. Klug

**Affiliations:** Lehrstuhl für Epidemiologie, Fakultät für Sport- und Gesundheitswissenschaften, Technische Universität München, Georg-Brauchle-Ring 56, 80992 München, Deutschland

**Keywords:** Humane Papillomviren, Impfung, Präkanzeröse Läsionen, Genitalwarzen, Zervixkarzinom, Human papillomavirus, Vaccination, Precancerous lesions, Genital warts, Cervical cancer

## Abstract

Humane Papillomviren (HPV) verursachen benigne und maligne Tumorerkrankungen. Bisher wurden mehr als 200 HPV-Typen entdeckt, von denen aktuell 12 als Hochrisiko für das Zervixkarzinom klassifiziert werden. HPV-Typen, die den Anogenitaltrakt befallen, werden sexuell übertragen. Seit 2006 sind prophylaktische HPV-Impfstoffe erhältlich. Die Impfung soll vor dem ersten sexuellen Kontakt erfolgen.

HPV infizieren Epithelzellen und sind die häufigsten sexuell übertragenen Viren weltweit. Neben dem Zervixkarzinom verursachen HPV auch andere anogenitale Tumore, wie Vulva‑, Vagina- und Analkarzinom, aber auch Oropharynxkarzinome. Vor allem die Hochrisiko-HPV-Typen 16 und 18 werden hier gefunden. Des Weiteren sind die HPV-Typen 6 und 11 ursächlich für die Entstehung von genitalen Warzen. Aber auch harmlose Hautwarzen werden von HPV verursacht.

HPV-Impfstoffe sind sicher und hochwirksam, wenn vor der Impfung noch keine HPV-Infektion vorliegt. Systematische Reviews und Metaanalysen haben gezeigt, dass die HPV-Impfung eine HPV-Infektion, aber auch präkanzeröse Läsionen im Anogenitaltrakt und Genitalwarzen wirksam verhindert. Mittlerweile liegen erste direkte Hinweise vor, dass die HPV-Impfung die Inzidenz des Zervixkarzinoms reduziert.

Die Impfquoten variieren weltweit je nach Impfprogramm und Akzeptanz des HPV-Impfstoffs in der Bevölkerung. Deutschland hat im Vergleich zu anderen europäischen Ländern niedrige Impfquoten. Die Ständige Impfkommission (STIKO) empfiehlt die HPV-Impfung in Deutschland für alle Mädchen und Jungen im Alter von 9 bis 14 Jahren. Im Jahr 2018 waren nur die Hälfte aller 18-jährigen Mädchen in Deutschland vollständig gegen HPV geimpft.

Organisierte Impfprogramme, bevölkerungsbezogen oder schulbasiert, sind notwendig, um hohe Impfquoten zu erreichen.

## Abstract (en)

Human papillomaviruses (HPVs) can cause both benign and malignant tumors. To date, more than 200 HPV types have been discovered, of which 12 are currently classified as high risk for cervical cancer. HPV types that affect the anogenital tract are sexually transmitted. Since 2006, prophylactic HPV vaccines have been available and should be administered before first sexual contact.

HPVs infect epithelial cells and are worldwide the most common sexually transmitted viruses. Apart from cervical cancer, HPVs cause other anogenital cancers such as vulvar, vaginal, and anal cancer but also oropharyngeal cancer (or head and neck cancers). HPV types 16 and 18 are also found at these sites. HPV types 6 and 11 are associated with genital warts; other HPV types can cause harmless skin warts.

HPV vaccines are safe and highly effective, if they are administered before exposure to HPV. Systematic reviews and meta-analyses have shown that HPV vaccination effectively prevents HPV infection, but also precancerous lesions of the anogenital tract and genital warts. Recent vaccination data also demonstrate reductions in cervical cancer incidence.

The uptake rates of HPV vaccination vary worldwide by program and acceptance. In comparison to other European countries, Germany has a low uptake rate. The Standing Committee on Vaccination (STIKO) recommends HPV vaccinations for all girls and boys ages 9 to 14 years in Germany. In 2018, only half of all 18-year-old girls in Germany were completely immunized against HPV.

Organized vaccination programs, whether population-based or school-based, are necessary in order to increase vaccination uptake rates.

## Hintergrund

Der kausale Zusammenhang zwischen humanen Papillomviren (HPV) und dem Zervixkarzinom eröffnete die Möglichkeiten einer Impfprävention. Im Jahr 2006 hat die Food and Drug Administration (FDA) in den USA den ersten HPV-Impfstoff (Gardasil®) zugelassen, weitere Zulassungsbehörden in Europa und weltweit folgten. Zwischenzeitlich wurden 2 weitere Impfstoffe gegen HPV (Cervarix® und Gardasil 9®) von der FDA und der europäischen Zulassungsbehörde (EMA) zugelassen und in vielen Ländern weltweit wurden HPV-Impfprogramme implementiert. Seitdem wurden viele Studien zur Wirkung der HPV-Impfung auf HPV-bedingte Erkrankungen veröffentlicht. Ziel dieser Übersicht ist es, eine Einführung zu HPV und HPV-bedingten Erkrankungen sowie zur HPV-Impfung zu geben und die vorhandene Evidenz zur Prävention von Genitalwarzen und Krebsvorstufen vorzustellen.

## Epidemiologie von HPV-Infektionen und Risikofaktoren

HPV sind DNA-Viren der Familie der Papillomaviridae und zählen zu den häufigsten sexuell übertragbaren Viren weltweit [1, 2]. Fast alle sexuell aktiven Menschen infizieren sich mit HPV, meistens bei den ersten sexuellen Kontakten. Die meisten HPV-Infektionen sind innerhalb von 2 Jahren nicht mehr nachweisbar, nur bei circa 10 % der Betroffenen persistiert die Infektion länger und kann zur Entstehung von präkanzerösen Läsionen führen [3]. Ein geringer Teil dieser Läsionen kann schlimmstenfalls zu einer HPV-bedingten Tumorerkrankung führen.

Bisher wurden mehr als 200 HPV-Typen identifiziert, die aufgrund ihres krebserregenden Potenzials in Hochrisiko-(HR-) und Niedrigrisiko-(LR-)HPV-Typen eingeteilt werden [4]. Aktuell werden 12 HR-HPV-Typen als krebserregend eingestuft [4]. Bei fast allen Zervixkarzinomen wird eine HPV-Infektion nachgewiesen [5]. Andere Krebserkrankungen bei Frauen, einschließlich Tumoren der Vagina, der Vulva, des Anus, der Mundhöhle und des Oropharynx werden mit einer HPV-Infektion assoziiert [6]. Bei Männern werden HPV-Infektionen mit Tumoren des Anus, des Penis, der Mundhöhle und des Oropharynx in Verbindung gebracht [6].

### Risikofaktoren

Eine HPV-Infektion verläuft meist ohne Symptome. Die Hauptrisikofaktoren für eine HPV-Infektion sind eine hohe Anzahl von Sexualpartnern, ein junges Alter bei sexueller Aktivität und das Rauchen [7]. HPV-Infektionen sind unter immungeschwächten Personen häufiger zu finden, so z. B. bei Personen mit HIV-Infektion oder Zustand nach Organtransplantation [7]. Kondome bieten keinen sicheren Schutz gegen eine HPV-Infektion, da die Viren auch durch Hautkontakt übertragen werden können [8].

Ein geschwächtes Immunsystem sowie das Rauchen erhöhen das Risiko für eine persistierende HPV-Infektion. Risikofaktoren sind zudem Co-Infektionen mit anderen sexuell übertragbaren Pathogenen, wie z. B. Herpes-simplex-Viren (HSV; [9]). Eine Metaanalyse ergab, dass die HPV-Typen 16, 31, 33 und 52 weltweit häufig bei persistierenden HPV-Infektionen gefunden werden [10]. Andere Untersuchungen haben gezeigt, dass HPV-Infektionen mit zunehmendem Alter häufiger persistieren [11].

### HPV-Prävalenz

Die Prävalenz von HPV-Infektionen variiert weltweit je nach geografischer Region, Alter, Geschlecht und untersuchter Studienpopulation. Eine Metaanalyse schätzte die globale genitale HPV-Prävalenz bei Frauen mit normaler Zytologie auf 11,7 % (95 % KI: 11,6–11,7 %), wobei die höchsten Prävalenzen in Subsahara-Afrika mit 24,0 %, in Osteuropa mit 21,4 % und in Lateinamerika mit 16,1 % gefunden wurde [12]. Die altersspezifische Prävalenz war bei unter 25-jährigen Frauen am höchsten [12]. In Deutschland schätzte eine Studie die HR-HPV-Prävalenz für Frauen ab 30 Jahren auf 7,1 % in Hannover und 5,9 % in Tübingen [13]. Bei Frauen unter 30 Jahren gehen Schätzungen von einer HPV-Prävalenz von 28,3 % aus [14].

Ein systematischer Review ergab sehr große Unterschiede in der globalen anogenitalen HPV-Prävalenz bei Männern [15]. Die geschätzten Prävalenzen an verschiedenen anatomischen Stellen lagen zwischen 1,3 % und 72,9 % [15]. Die HPV-Prävalenz am Penis lag zwischen 5,6 % und 51,5 %, am Skrotum zwischen 7,1 % und 46,2 % in Abhängigkeit von der Studienpopulation und der HPV-Testmethode [15]. Eine US-amerikanische Querschnittsstudie ergab eine genitale HPV-Prävalenz bei Männern von 45,8 % (95 % KI: 41,9–49,7) und HR-HPV von 25,7 % (95 % KI: 23,4–28,1; [16]). Die orale HPV-Prävalenz bei Männern wurde auf 11,5 % geschätzt [17].

## HPV-assoziierte Krankheiten

**Figure 1:**
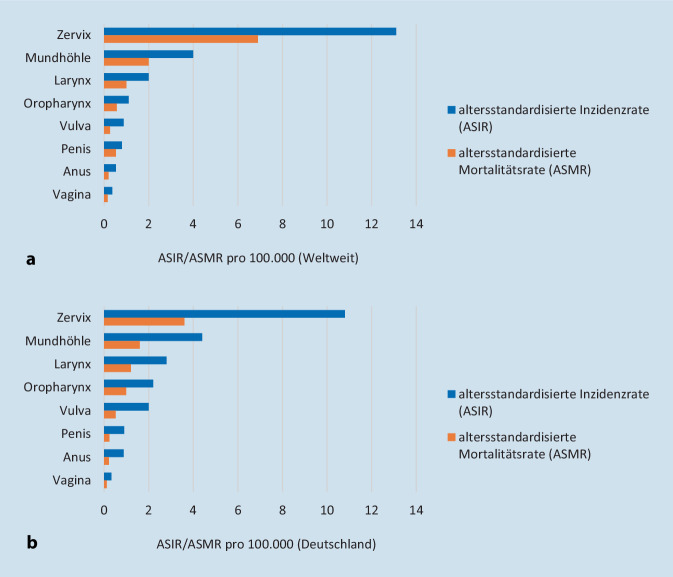

HPV infiziert Epithelzellen am ganzen Körper und kann benigne und maligne Tumore verursachen. Die HPV-Typen 1, 2, 3, 4, 10 verursachen harmlose Hautwarzen, beispielweise an den Füßen, vor allem bei Kindern [7, 18]. Die HPV-Typen 6 und 11 sind als Niedrigrisiko eingestuft und verursachen genitale Warzen [4, 19]. Aktuell werden 12 HPV-Typen (HPV 16, 18, 31, 33, 35, 39, 45, 51, 52, 56, 58, 59) als Hochrisiko für die Entstehung eines Zervixkarzinoms eingestuft [4, 19]. Einige Hochrisiko-HPV-Typen, besonders HPV-Typ 16, sind mit hochgradigen intraepithelialen Neoplasien im Anogenitaltrakt assoziiert, die zu malignen Tumoren führen können [7, 19]. In Abb. 1 sind Inzidenz- und Mortalitätsschätzungen von HPV-assoziierten Tumorentitäten weltweit und in Deutschland dargestellt [20].

### Krebsvorstufen

Die meisten HPV-Infektionen bilden sich ohne Behandlung spontan zurück, während ein geringerer Prozentsatz persistiert [21]. Persistierende HPV-Infektionen an der Cervix uteri (Gebärmutterhals) sind mit der Entwicklung von Krebsvorstufen, den sogenannten CIN-Läsionen (zervikale intraepitheliale Neoplasien, ICD-10 N87) assoziiert. Im Stadium CIN 1 liegen milde Veränderungen (Dysplasien) vor, die sich häufig von alleine zurückbilden. Je weiter die Vorstufen progredieren, umso unwahrscheinlicher wird eine spontane Ausheilung [21]. Ein systematischer Literaturreview mit Metaanalyse ergab, dass sich bei einer Nachbeobachtungszeit von 24 Monaten die Hälfte der CIN 2 zurückbilden und ca. 30 % persistieren, während sich ungefähr ein Fünftel weiter zu einer CIN-3-Läsion entwickelt [22].

Persistierende HPV-Infektionen sind auch mit intraepithelialen Neoplasien der Vulva (VIN, N90), der Vagina (VAIN, N89), des Penis (PIN) und des Anus bzw. der Perianalhaut (AIN, PAIN) assoziiert [19]. Diese werden in niedriggradige (VIN 1, PIN 1, AIN 1) und hochgradige Läsionen (VIN 2–3, PIN 2–3, AIN 2–3) unterteilt, die Haut und Schleimhaut betreffen können, die aus Plattenepithel besteht [19]. Unbehandelt können sich diese Vorstufen zu Karzinomen entwickeln.

Die Behandlung von HPV-assoziierten Krebsvorstufen, die im Anogenitaltrakt beider Geschlechter auftreten können, belastet auch das Gesundheitssystem [7, 19].

### Zervixkarzinom

HR-HPV-Infektionen sind ursächlich für die Entstehung eines Zervixkarzinoms [5]. Das invasive Zervixkarzinom (ICD-10 C53) ist eine der häufigsten Krebsarten bei Frauen weltweit, mit einer altersstandardisierten Inzidenzrate (ASIR, Weltstandard) von 13,1 pro 100.000 und einer altersstandardisierten Mortalitätsrate (ASMR) von 6,9 pro 100.000 im Jahr 2018 [23]. Im selben Jahr waren Zervixkarzinome die vierthäufigste Todesursache weltweit und gehörten in 146 (79 %) von 185 Ländern zu den 3 häufigsten Krebsarten bei Frauen unter 45 Jahren [23]. Die höchsten Inzidenz- und Mortalitätsraten werden in weniger entwickelten Ländern beobachtet, mit einem Anteil von 84 % aller Zervixkarzinome und 88 % der Todesfälle durch Zervixkarzinom im Jahr 2018 [23].

In den Industrienationen sind die Inzidenz und Mortalität des Zervixkarzinoms im Laufe der Jahre aufgrund von Screeningmaßnahmen zurückgegangen. Die ASIR (Europa Standard) lag im Jahr 2016 bei 8,7 pro 100.000, wobei die höchste Inzidenz bei 40- bis 44-jährigen Frauen gefunden wird (16,6 pro 100.000, rohe Rate; [24]). Nach Schätzungen von 2018 weist Deutschland nach Belgien eine der höchsten Inzidenzraten in Westeuropa auf [25].

Deutschland hat seit 1971 ein opportunistisches Zervixkarzinomscreening, das 2020 durch ein organisiertes Programm ersetzt wurde. Bis 2019 hatten alle Frauen ab 20 Jahren einen Anspruch auf eine jährliche zytologische Screeninguntersuchung mit dem Pap-Abstrich. Im neuen organisierten Programm erhalten junge Frauen im Alter von 20 bis 34 Jahren weiterhin ein jährliches zytologisches Screening und Frauen ab 35 Jahren erhalten alle 3 Jahre eine Co-Testung mit Pap-Abstrich und HPV-Test. Die Krankenkassen verschicken zudem alle 5 Jahre ein Informationsschreiben zum Programm an alle berechtigten Frauen bis 65 Jahre. Das neue Programm wird nach 6 Jahren einer Evaluation unterzogen [26].

### Andere anogenitale Krebserkrankungen

Auch die Krankheitslast durch andere HPV-assoziierte Tumorerkrankungen ist sehr hoch. Im Jahr 2012 waren weltweit 25 % der Vulva-(C51), 78 % der Vaginal-(C52), 88 % der Anal-(C21) und 50 % der Peniskarzinome (C60) auf eine HPV-Infektion zurückzuführen. Die höchsten Inzidenzraten wurden in Latein- und Nordamerika sowie in Australien verzeichnet [6]. Zwar treten diese Krebsentitäten in der Bevölkerung selten auf, allerdings gibt es z. B. beim Analkarzinom Risikogruppen mit sehr hohen Inzidenzen, wie etwa Männer, die Sex mit Männern haben (MSM; [27]). Für Deutschland schätzte eine Studie für 2013 eine ASIR für anogenitale Krebserkrankungen mit HPV-bedingter Morphologie von 2,3 (95 % KI: 2,2–2,4) für Männer und 14,8 (95 % KI: 14,4–15) für Frauen [28].

### Kopf- und Halstumoren

Im Jahr 2012 wurden 30,8 % der Oropharynxkarzinome (C01, C09–10), 2,2 % der Mundhöhlekarzinome (C02–06) und 2,4 % der Larynxkarzinome (C32) auf HPV zurückgeführt, wobei die Anteile in Nordamerika und Europa im weltweiten Vergleich höher waren [6]. Die Anteile der HPV-Typen 16 und 18 an allen HPV-attributablen Kopf- und Halstumoren lag im Jahr 2018 bei 85 % weltweit [6].

### Genitale Warzen

Die LR-HPV-Typen 6 und 11 sind mit ca. 90 % der Genitalwarzen (A63.0; [29]), einer der häufigsten sexuell übertragbaren Krankheiten weltweit, assoziiert [30]. Bei Frauen können Genitalwarzen an Vulva, Vagina oder Anus auftreten, bei Männern am Penis, Anus oder am Skrotum [31]. Die Prävalenz von Genitalwarzen variiert weltweit zwischen 0,13 % und 5 %, wobei die höchsten Raten bei Jugendlichen und jungen Erwachsenen zu verzeichnen sind [30].

Die Behandlungen von Genitalwarzen ist oft langwierig und sowohl für die Betroffenen als auch das Gesundheitssystem belastend [7, 19]. Eine bevölkerungsbezogene australische Studie schätzte die Kosten für die Behandlungen von Genitalwarzen auf über 14 Mio. australische Dollar im Jahr [32]. Eine deutsche Studie schätzte die Kosten für eine Behandlung von Genitalwarzen auf 400 € bis über 1000 € pro Patient [33].

## Die HPV-Impfung weltweit

Seit den 1970er-Jahren hat der deutsche Virologe Harald zur Hausen eine Verbindung zwischen HPV und Zervixkarzinom hergestellt [34, 35]. Bis 1980 identifizierten er und sein Team die HPV-Typen 16 und 18 in Zervixkarzinombiopsien [36]. In den 1990er-Jahren bestätigten dann eine Reihe epidemiologischer Fallkontrollstudien und später auch Kohortenstudien den Zusammenhang zwischen einer HPV-Infektion und der Entstehung des Zervixkarzinoms [37, 38]. Im Jahr 2006 wurde der erste HPV-Impfstoff (Gardasil®4) von der FDA in den USA zugelassen. 2 Jahre später wurde Harald zur Hausen der Nobelpreis für Medizin verliehen.

Bisher wurden 3 Impfstoffe von der FDA zugelassen: Gardasil®4, Cervarix® und Gardasil®9 (Tab. 1; [39, 40]). Alle 3 Impfstoffe schützen vor den HPV-Typen 16 und 18, die bis zu 70 % der Zervixkarzinome verursachen. Gardasil®9 bietet zusätzlichen Schutz vor einer Infektion mit 5 weiteren HR-HPV-Typen (31, 33, 45, 52, 58), die weitere 20 % der Zervixkarzinome verursachen [39]. Beide Gardasil®-Impfstoffe immunisieren zudem gegen Infektionen mit den LR-HPV-Typen 6 und 11, die mit Genitalwarzen assoziiert sind [39]. Es wird erwartet, dass die HPV-Impfstoffe auch andere HPV-assoziierte anogenitale Tumore sowie Kopf- und Halstumore verhindern. Allerdings entfalten die HPV-Impfstoffe ihre höchste Wirksamkeit nur, wenn die Impflinge bei Impfung HPV-naiv sind, also noch keine Infektion mit HPV vorliegt. Daher sollen Mädchen und Jungen vor den ersten sexuellen Kontakten geimpft werden.

**Table 1:** 

Impfstoffbezeichnung	Schutz vor HPV-Typ	Zulassungsdatum
Gardasil®	6, 11, 16, 18	20.09.2006
Cervarix®	16, 18	20.09.2007
Gardasil® 9	6, 11, 16, 18, 31, 33, 45, 52, 58	10.06.2015

Die HPV-Impfstoffe gelten als wirksam und sicher. Das Risiko von Nebenwirkungen ist gering [41]. Die HPV-Impfung wurde zuerst für Mädchen zum Schutz vor dem Zervixkarzinom empfohlen. Es wurde berichtet, dass der HPV-Impfstoff bis zu 99 % der HPV-Infektionen und fast alle zervikalen Vorstufen verhindert, wenn bei der Impfung noch keine HPV-Infektion vorlag [41]. Bisher liegen Daten vor, die zeigen, dass die Wirkung des Impfstoffs mindestens 14 Jahre anhält [42].

Mittlerweile wird die HPV-Impfung auch für Jungen empfohlen. Schätzungen zeigen eine Wirksamkeit von mehr als 96 % gegen persistierende anogenitale HPV-18-Infektionen bei HPV-naiven Männern [43]. Modellstudien zeigen, dass die Impfung von Jungen die Belastung durch HPV-Infektionen und auch die Inzidenz des Zervixkarzinoms und anderer anogenitaler Krebserkrankungen weiter verringert [7, 44].

### HPV-Impfprogramme

Bis zum Jahr 2019 haben nach Angaben der Weltgesundheitsorganisation (WHO) 106 Länder HPV-Impfprogramme eingeführt [45]. Dennoch decken diese Programme bislang nur ein Drittel der Mädchen zwischen 9 und 14 Jahren weltweit ab [45]. Trotz der im Laufe der Jahre gestiegenen Impfquoten treten 61 % der Fälle von Gebärmutterhalskrebs in Ländern auf, in denen die HPV-Impfung noch nicht eingeführt wurde [45].

Die HPV-Impfquoten im Jahr 2018 zeigen große Unterschiede zwischen den Ländern weltweit. In Tab. 2 sind die Impfquoten in der Europäischen Union aufgeführt. Die höchsten Impfquoten sind bei 15-jährigen Mädchen in Portugal (95 %) zu verzeichnen, gefolgt von Island (88 %) und Norwegen (85 %; [45]). Die hohen Impfquoten in Portugal können auf die große Akzeptanz von Impfstoffen in der Bevölkerung zurückgeführt werden. Norwegen und Island haben die hohen Impfquoten durch schulbasierte Impfprogramme erzielt. Auch England (Vereinigtes Königreich), Spanien und Schweden haben im Rahmen von Schulimpfprogrammen Impfquoten über 80 % erzielt (Tab. 2). Die meisten europäischen Länder impfen im Alter von 12 bzw. 13 Jahren gegen HPV.

**Table 2:** 

Land	Einführungsjahr	Art des HPV-Impfprogramms	Impfalter (Jahre)	Erste Dosisabdeckung^b^ (in %)	Letzte Dosisabdeckung^c^ (in %)	Vollständige Impfquote bei 15-Jährigen^d^	
						Mädchen (in %)	Jungen (in %)
Belgien	2007	Schulimpfprogramm	12–14	71	67	67	NA
Dänemark	2009	Gesundheitszentren	12	79	62	77	NA
Deutschland	2007	Niedergelassene Ärzte	9–14	48	31	31	NA
Estland	2018	Schulimpfprogramm	12–14	51	45	48	Nicht eingeführt
Finnland	2013	Schulimpfprogramm	11–12	NA	60	NA	Nicht eingeführt
Frankreich	2007	Gesundheitszentren	11–14	29	24	24	Nicht eingeführt
Irland	2010	Schulimpfprogramm	12–13	85	69	60	NA
Island	2011	Schulimpfprogramm	12	94	85	88	Nicht eingeführt
Italien	2008	Gesundheitszentren	11	61	40	67	8
Lettland	2010	Schulimpfprogramm und Gesundheitszentren	12	NA	54	39	Nicht eingeführt
Litauen	2016	NA	11	NA	66	NA	Nicht eingeführt
Luxemburg	2008	Gesundheitszentren	9–13	37	14	43	NA
Malta	2012	Gesundheitszentren	12	86	81	82	Nicht eingeführt
Niederlande	2009	Gesundheitszentren^e^	12–13	63	53	51	Nicht eingeführt
Norwegen	2009	Schulimpfprogramm	12	98	91	85	NA
Portugal	2008	Gesundheitszentren	13	93	81	95	Nicht eingeführt
Schweden	2012	Schulimpfprogramm	10–12	86	80	75	Nicht eingeführt
Schweiz	2007	Gesundheitszentren	11–14	64	59	59	17
Slowenien	2009	Schulimpfprogramm	11–12	NA	59	40	Nicht eingeführt
Spanien	2007/2008	Schulimpfprogramm oder Gesundheitszentren (je nach Region)	12	84	79	80	Nicht eingeführt
Ungarn	2014	Schulimpfprogramm	12	86	78	72	Nicht eingeführt
Vereinigtes Königreich	2008–2012	Schulimpfprogramm	11–13	85	82	81	NA
Zypern	2016	Schulimpfprogramm	12–13	73	64	59	Nicht eingeführt

*NA* Keine Daten

^a^Nur Länder, für die Impfstatistiken verfügbar waren

^b^Zielgruppe, die 2018 die erste Dosis HPV-Impfstoff erhalten hat

^c^Zielgruppe, die 2018 die letzte Dosis HPV-Impfstoff erhalten hat

^d^Bevölkerung, die 2018 15 Jahre alt wurde und jederzeit zwischen 9 und 14 Jahren eine vollständige Impfung erhielt

^e^Alle Mädchen erhalten eine Einladung, wenn sie 13 Jahre alt werden

Die Impfquoten bei 15-jährigen Mädchen in Deutschland waren niedriger (31 %) als in den meisten europäischen Ländern, nur in Frankreich (24 %) fielen die Impfquoten noch geringer aus [45]. Beide Länder haben kein organisiertes oder schulbasiertes Impfprogramm.

## HPV-Impfung in Deutschland

Die HPV-Impfung ist seit Ende 2006 in Deutschland erhältlich und wurde von der Ständigen Impfkommission (STIKO) im Jahr 2007 zunächst für Mädchen im Alter von 12 bis 17 Jahren empfohlen. Im Jahr 2014 wurde die Empfehlung angepasst und das Impfalter auf 9 bis 14 Jahre herabgesetzt [46]. Seit 2018 wird die Impfung auch für Jungen im Alter 9 bis 14 Jahre empfohlen [47]. Für diese Altersgruppe wird eine Impfung mit 2 Dosen im Abstand von 5 Monaten empfohlen, für Jugendliche über 14 Jahre ist eine Impfung mit 3 Dosen erforderlich [46, 47]. Nachholimpfungen werden bis zum 17. Lebensjahr empfohlen [47]. Auch immunsupprimierte Personen können möglicherweise von einer HPV-Impfung profitieren [48]. In Infobox 1 sind wichtige Informationen zur HPV-Impfempfehlung für Deutschland zusammengefasst.

### Infobox 1 Wichtige Informationen zur HPV-Impfung. Adaptiert nach der HPV-Impfempfehlung der Ständigen Impfkommission (STIKO; [46, 47])

*Wer?* Mädchen und Jungen

*Wann?* Im Alter von 9 bis 14 Jahren

*Wie oft?* 2 Impfungen im Abstand von mindestens 5 Monaten

*Wo?* Niedergelassene Kinderärzte, Allgemeinärzte, Gynäkologen, Urologen

*Wichtig!* Die Impfung soll vor dem ersten sexuellen Kontakt abgeschlossen sein

**Figure 2:**
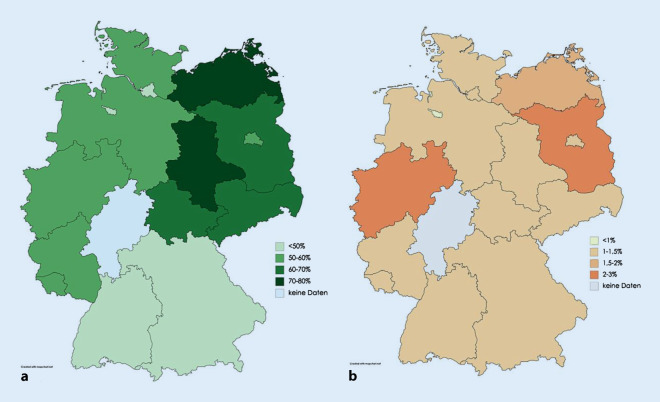

In Abb. 2 sind die Impfquoten bei 18-jährigen Mädchen und Jungen mit einer vollständigen Impfung nach Bundesländern im Jahr 2018 dargestellt. In diesem Jahr wurden 51,1 % der 18-jährigen Mädchen in Deutschland vollständig geimpft [49]. Die höchste HPV-Impfquote bei 18-jährigen Mädchen wurde in Sachsen-Anhalt (71,7 %) erzielt, die niedrigste in Bremen (40,7 %; [49]). Im selben Jahr wurden in Deutschland nur 1,3 % der 18-jährigen Jungen vollständig geimpft [49].

In Deutschland ist bisher kein organisiertes HPV-Impfprogramm eingeführt worden. Mädchen und Jungen können kostenlos bei niedergelassenen Ärzten geimpft werden. HPV-Impfungen erfolgen vor allem bei niedergelassenen Kinderärzten, aber auch bei Allgemeinärzten, Gynäkologen und Urologen. Eine Querschnittsstudie im Jahr 2010 mit Schülerinnen und Schülern der 10. Klasse in Berlin zeigte, dass Ärzte und Eltern eine wichtige Rolle bei HPV-Impfentscheidungen spielten [50]. Von den ungeimpften Mädchen gaben 30,8 % an, sich aufgrund von Bedenken hinsichtlich der Nebenwirkungen des Impfstoffs nicht impfen lassen zu wollen [50]. Eine andere im Jahr 2010 in Berlin durchgeführte Studie mit 18- bis 25-jährigen Auszubildenden zeigte, dass das Wissen über HPV sehr gering war [51]. 51 % der jungen Frauen und 42 % der jungen Männer waren der Meinung, dass sich nur Frauen infizieren werden [51]. Wissenschaftlich fundierte Impfkampagnen, wie beispielsweise durch die Bundeszentrale für gesundheitliche Aufklärung (BZgA), können, wie auch engagierte Aufklärung in der Schule, dazu beitragen, dass Heranwachsende besser über HPV und die HPV-Impfungen informiert werden.

## Einfluss der HPV-Impfung auf Krebsvorstufen und Genitalwarzen

Nach der Einführung der HPV-Impfung wurden viele Studien durchgeführt, um die Auswirkungen der Impfung auf die Entstehung anogenitaler Erkrankungen zu bewerten. Es wurden mehrere systematische Reviews und Metaanalysen veröffentlicht, um die Evidenzen dieser Studien zusammenzufassen. Die Hauptendpunkte waren zunächst Genitalwarzen und präkanzeröse Läsionen der Cervix uteri.

Die ersten Evidenzen für die Wirkung der HPV-Impfstoffe stammen aus Australien, einem der ersten Länder, welches früh ein nationales HPV-Impfprogramm eingeführt hat. Australien hat HPV-Impfquoten von ca. 80 % erreicht und ein nationales Surveillance-System eingeführt, um die Auswirkungen der Impfung zu überwachen [52]. Die hohen Impfquoten wurden durch die Einführung eines schulbasierten HPV-Impfprogramms erreicht, in dessen Rahmen seit dem Jahr 2007 Mädchen und seit 2013 auch Jungen im Alter zwischen 12 und 13 Jahren geimpft werden.

Kurz nach der Einführung der HPV-Impfung in Australien zeigte sich bereits ein Rückgang bei Krebsvorstufen und Genitalwarzen. Brotherton und Kollegen haben bei Mädchen unter 18 Jahren einen Rückgang der hochgradigen Läsionen (CIN2+) nach Einführung des Impfstoffs (im Vergleich zum Zeitraum vor der Impfung) auf 0,38 % (95 % KI: 0,61–0,16) geschätzt [53]. Diese ökologische Studie zeigte keine signifikante Abnahme der zervikalen Läsionen bei Frauen über 18 Jahren.

Unter Verwendung der Surveillance-Daten zeigten Donovan und Kollegen eine signifikante Abnahme der Diagnose von Genitalwarzen 3 Jahre nach Einführung des HPV-Impfprogramms bei Frauen im Alter von 12 bis 26 Jahren (59 %, Trend < 0,0001) und heterosexuellen Männern im gleichen Alter (39 %, Trend < 0,0001), jedoch nicht für Personen über 26 Jahre und MSM [52]. Hiermit wurden auch die ersten Auswirkungen einer Herdenimmunität gezeigt. Eine weitere australischen Studie zeigte eine Abnahme des Anteils der Genitalwarzen bei Frauen unter 21 Jahren von 18,4 % (2004/2005) auf 1,1 % (2013/2014; [54]). Bei heterosexuellen Männern unter 21 Jahren gab es eine Abnahme der Diagnose von Genitalwarzen im gleichen Zeitraum von 11,3 % auf 2,8 % [54].

Studien aus anderen Ländern haben einen ähnlichen Rückgang der Inzidenz von Krebsvorstufen und Genitalwarzen berichtet. Eine ökologische Studie in England (Vereinigtes Königreich) zeigte einen Rückgang der Genitalwarzen bei Mädchen im Alter von 15 bis 19 Jahren zwischen 2009 und 2014 um 30,6 % [55]. Der größte Rückgang konnte mit 50,9 % bei 15-jährigen Mädchen beobachtet werden [55]. Der jährliche Rückgang der Inzidenz von Genitalwarzen bei Frauen zwischen 2009 und 2015 betrug 4,8 % (95 % KI: 4,3–5,3) in Norwegen und 18 % (95 % KI: 17,5–18,6) in Dänemark [56]. Eine neuere Studie, unter Verwendung von Registerdaten aus Dänemark zeigte, dass nach Einführung der HPV-Impfung die Inzidenz von VIN (bei Frauen < 20 und 20–29 Jahre alt) im Vergleich zu den Jahren zuvor abnahm [57]. Alle drei Länder haben hohe HPV-Impfquoten erreicht (Tab. 2).

Mittlerweile wurden erste Auswirkungen der HPV-Impfung auf die Inzidenz von Gebärmutterhalskrebs berichtet. Eine Registerstudie in Schweden, die Mädchen und Frauen im Alter von 10 bis 30 Jahren von 2006 bis 2017 untersuchte, konnte zeigen, dass das Risiko für Zervixkarzinome bei geimpften Frauen im Vergleich zu nicht geimpften Frauen um 63 % niedriger war (0,37, 95 % KI: 0,21–0,57; [58]). Die höchste Risikoreduktion (88 %) wurde bei Frauen beobachtet, die vor dem 17. Lebensjahr geimpft wurden (0,12, 95 % KI: 0,00–0,34).

Für Deutschland liegen bisher nur wenige Daten vor. Eine bevölkerungsbezogene Querschnittsstudie berichtete über eine signifikant niedrigere Prävalenz von HPV 16 und 18 bei geimpften Frauen im Alter von 20 bis 25 Jahren im Vergleich zu nicht geimpften Frauen im gleichen Alter [59]. Nach Subgruppenanalyse war dieses Ergebnis jedoch nur für die Altersgruppe 20 und 21 statistisch signifikant [59].

Eine weitere Studie unter Verwendung von Versichertendaten von ca. 60.000 jungen Frauen zeigte einen signifikanten Rückgang von zervikalen Läsionen und Genitalwarzen [60]. Die Prävalenz von Genitalwarzen lag für die Geburtskohorte 1989 bei 1,30 % (95 % KI: 1,12–1,49), während in jüngeren Geburtskohorten die Prävalenz niedriger war (0,94, 95 % KI: 0,79–1,10, Geburtskohorte 1992; [60]).

### Evidenzen von systematischen Reviews und Metaanalysen

Die Ergebnisse eines systematischen Reviews mit Metaanalyse zeigten eine signifikante Reduktion der Prävalenz der HPV-Typen 16 und 18 bei 15- bis 24-jährigen Frauen 4 Jahre nach der Impfung [61]. Bei 25- bis 29-jährigen Frauen wurde eine Reduktion erst 5 bis 8 Jahre nach Einführung der HPV-Impfung festgestellt. Das Auftreten von genitalen Warzen war nach dem gleichen Zeitrahmen bei 15- bis 29-jährigen Frauen und 15- bis 24-jährigen Männern signifikant reduziert [61]. Bei Frauen im Alter von 15 bis 24 Jahren wurde eine signifikante Reduktion von CIN2+ berichtet, während bei älteren nicht geimpften Frauen ein Anstieg beobachtet wurde. Die höchsten Reduktionen waren in Ländern mit hohen Impfquoten (≥ 50 %) beobachtbar, die mehrere Jahrgänge geimpft hatten [61].

Ein Umbrella-Review, der Evidenzen aus verschiedenen systematischen Reviews zusammenfasste, kam zu dem Schluss, dass eine HPV-Impfung Schutz vor Genitalwarzen und präkanzerösen Läsionen bei Männern und Frauen bietet [62]. Das Risiko für Genitalwarzen bei geimpften Personen war deutlich niedriger und lag zwischen 0,05 (95 % KI: 0,01–0,25) und 0,38 (95 % KI: 0,31–0,47). Die Risikoschätzungen für präkanzeröse Läsionen reichten von 0,8 (95 % KI: 0,62–1,02) für CIN 2+ zu 0,01 (95 % KI: 0,0–0,1) für CIN 3+ [62].

### Daten aus Modellierungsstudien

Modellierungsstudien prognostizieren eine deutliche Reduktion sowohl der zervikalen Läsionen als auch der Inzidenz des Zervixkarzinoms. Basierend auf einem systematischen Review mit einer Metaanalyse von Modellierungsstudien ist die Eliminierung der HPV-Typen 6, 11, 16 und 18 mit einer Impfquote von 80 % bei Mädchen und Jungen möglich [63].

In einer kürzlich durchgeführten Modellierungsstudie wurde geschätzt, dass, wenn 90 % aller Mädchen im Alter von 15 Jahren geimpft würden, 70 % der Frauen zweimal im Leben (Alter 35 und 45) gescreent würden und 90 % der präkanzerösen zervikalen Läsionen behandelt würden, eine Reduktion der weltweiten altersstandardisierten Inzidenz des Zervixkarzinoms auf 4 pro 100.000 möglich wäre [64]. Dies unterstreicht die Notwendigkeit, die Impfquoten bei Mädchen und Jungen sowie die Screeningteilnahme bei Frauen deutlich zu erhöhen sowie Behandlungsstrategien umzusetzen, insbesondere in weniger entwickelten Ländern, in denen die meisten Zervixkarzinome auftreten. Die WHO hat im November 2020 eine neue globale Strategie zur Eliminierung des Zervixkarzinoms gestartet; dabei sind Impf- und Screeningprogramme die Schlüsselfaktoren [65].

In einer anderen Studie wurde geschätzt, dass der nonavalente (neunfach) Impfstoff (Gardasil®9) zu einer weiteren Senkung der hochgradigen Krebsvorstufen um 35 % beitragen würde [66]. Die Mehrzahl der Studien hat bisher allerdings über die Wirksamkeit von Gardasil®4 und Cervarix® berichtet, da der nonavalente Impfstoff erst seit 2015 erhältlich ist.

### Limitationen und zukünftige Perspektiven

Zusammenfassend zeigen die Evidenzen eine deutliche Abnahme der zervikalen Läsionen und genitalen Warzen bei Frauen, die vor den ersten sexuellen Kontakten geimpft wurden. Da auch über eine Reduktion der zervikalen Läsionen bei älteren Geburtskohorten von Frauen und über eine Reduktion der Genitalwarzen bei heterosexuellen Männern berichtet wurde, ist anzunehmen, dass es sich hierbei um Auswirkungen der Herdenimmunität handelt.

Bisher gibt es noch nicht genügend Hinweise über die Auswirkungen der HPV-Impfung auf präkanzeröse Läsionen bei Männern. Viele Industrieländer haben in den letzten Jahren damit begonnen, Männer gegen HPV zu impfen. Dies wird voraussichtlich zu einer weiteren Reduktion der Rate von Genitalwarzen beitragen sowie auch langfristig zu einer Reduktion von HPV-assoziierten präkanzerösen Läsionen und Tumorentitäten bei Männern und Frauen führen. Außerdem wird dies dazu beitragen, die Inzidenz von Analkarzinomen zu reduzieren, die in Hochrisikogruppen wie MSM häufig auftreten. Hieraus wird deutlich, dass noch weitere langfristige Studien zu den Auswirkungen der HPV-Impfung bei Frauen und bei Männern durchgeführt werden sollten.

Aufgrund des ökologischen Designs einiger Studien, die mit aggregierten Daten arbeiten, wurden in diesen Studien der Impfstatus von einzelnen Personen sowie weitere individuelle Risikofaktoren für eine HPV-Infektion, wie z. B. sexuelles Verhalten oder Rauchen, nicht berücksichtigt. In diesem Kontext kann argumentiert werden, dass die HPV-Impfung nicht der einzige Faktor ist, der die Inzidenz von präkanzerösen Läsionen und Genitalwarzen beeinflusst. Allerdings wurde in den meisten Ländern bisher nicht über Änderungen von Risikofaktoren in der Bevölkerung (z. B. Anzahl der Sexualpartner) berichtet. Zudem haben auch Studien, die den individuellen Impfstatus in ihren Analysen berücksichtigten, signifikante Effekte der HPV-Impfung nachgewiesen. Außerdem haben systematische Reviews eine hohe Wirksamkeit bei der Prävention von Genitalwarzen und präkanzerösen Läsionen der Cervix uteri gezeigt, sodass davon ausgegangen werden kann, dass die HPV-Impfung der Hauptfaktor für die Reduktion der Inzidenzraten ist.

Die meisten Schätzungen zur Wirksamkeit der HPV-Impfung stammen aus Industrieländern mit bestehenden Zervixkarzinomscreeningprogrammen. Die Teilnahme am Screening kann jedoch von Land zu Land variieren. Unterschiede in den Screeningprogrammen erschweren den Vergleich der Ergebnisse zwischen den verschiedenen Ländern. Einige Länder, einschließlich Deutschland, haben zudem keine systematische Registrierung der HPV-Impfung oder von Krebsvorstufen. Es ist daher entscheidend, ein geeignetes Surveillance-System zu implementieren, um die Wirkung der HPV-Impfung nachvollziehen und korrekt beurteilen zu können.

Die hohe Wirksamkeit der HPV-Impfung wird künftig Anpassungen in den Zervixkarzinomscreeningprogrammen erfordern. Bei geimpften Frauen können Screeningintervalle deutlich verlängert werden und damit die Anzahl der Screeninguntersuchungen deutlich reduziert werden. Hohe HPV-Impfquoten und ein organisiertes Zervixkarzinomscreening werden dazu beitragen, HPV-Infektionen, Krebsvorstufen und Gebärmutterhalskrebs deutlich zu reduzieren, vielleicht eines Tages sogar zu eliminieren.

## Fazit

Die HPV-Impfung zeigt sehr vielversprechende Ergebnisse hinsichtlich der Prävention von Genitalwarzen, Krebsvorstufen und dem Zervixkarzinom. Es sind jedoch hohe Impfquoten erforderlich, um einen sehr guten Schutz gegen HPV-bedingte Erkrankungen in der Bevölkerung zu erreichen. Niedrige Impfquoten sind auf das Fehlen von bevölkerungsbezogenen oder schulbasierten HPV-Impfprogrammen zurückzuführen. Für Deutschland sollte ein organisiertes oder schulbasiertes Programm für Mädchen und Jungen eingeführt werden, um eine Erhöhung der HPV-Impfquoten zu erreichen. Das neue organisierte Zervixkarzinomscreeningprogramm in Deutschland sollte unter Berücksichtigung der bereits HPV-geimpften Frauen künftig modifiziert werden.
